# Methodology for computational assessment of hemolysis in under‐occlusive roller pumps operated at clinically relevant conditions

**DOI:** 10.1002/btm2.70191

**Published:** 2026-11-10

**Authors:** Jiří Kohút, Jiří Jagoš, Pavel Suk, Martin Formánek, Jiří Burša

**Affiliations:** ^1^ Faculty of Mechanical Engineering Brno University of Technology Brno Czech Republic; ^2^ Department of Anaesthesia and Intensive Care St. Anne's University Hospital and Masaryk University Brno Czech Republic

**Keywords:** CFD simulations, extracorporeal circuits, hemolysis, roller pumps, shear stress, under‐occlusion

## Abstract

Hemolysis remains a major concern in clinical procedures involving extracorporeal blood circuits, with the blood delivery pump often being its considerable source. This study focuses on roller pumps, which—alongside centrifugal pumps—are the main types used in clinical practice. The objective is to establish a comprehensive methodology for the computational evaluation of hemolysis in under‐occlusive roller pumps, which, compared to fully occlusive operation, have the potential to reduce hemolysis while maintaining sufficient flow performance. The methodology employs transient 2D CFD simulations with a dynamic mesh, while hemolysis is evaluated using a shear‐induced hemolysis model. The governing equations are solved using the transitional low‐*Re* k−ω SST turbulence model with appropriate prism‐layer refinement at the wall, adhering to $y^{+}$ values lower than 1, which is essential for accurate hemolysis estimation. In contrast to existing studies, the simulations are complemented by experimental measurements from an in vitro circuit operated under clinically relevant settings, providing realistic boundary conditions and validation support. The core component of the circuit is an in‐house roller pump that enables precise setting and control of the under‐occlusion degree. The proposed methodology enables to estimate and compare hemolysis across five under‐occlusion settings with progressively increasing gap size, each characterized by the corresponding reduction in flow rate. The results indicate that hemolysis levels remain comparable across under‐occlusive settings with flow reductions of up to 37%, with no substantial decrease observed over this range. A more pronounced reduction in hemolysis is observed only at higher degrees of under‐occlusion, corresponding to 50% and 63% flow reductions, which, however, result in clinically unacceptable losses in flow performance. Thus, the simulations suggest that the tightest under‐occlusive setting with 10% flow reduction provides the most favorable balance between hemolysis and flow performance.

**Translational Impact Statement**

Compared to centrifugal pumps, computational studies on hemolysis in roller pumps remain scarce and methodologically incomplete. In particular, they lack an experimental counterpart, a crucial prerequisite for under‐occlusive simulations. To address this limitation, this article presents a comprehensive framework for hemolysis assessment that integrates high‐fidelity CFD approaches with experimentally derived boundary conditions and validation measurements. This enables simulations to be performed under realistic conditions and facilitates clinically meaningful comparison of hemolysis across different under‐occlusive settings.

## 1 INTRODUCTION

In clinical practice, roller pumps (RPs) are utilized in several procedures, including veno‐arterial extracorporeal membrane oxygenation (VA‐ECMO), cardio‐pulmonary bypass (CPB), and dialysis. As blood passes through a pump, erythrocytes are exposed to high stresses that can cause cellular damage and result in hemoglobin leakage, a condition known as hemolysis. Hemolysis induced during such clinical procedures poses a significant risk, particularly in prolonged cases of VA‐ECMO therapy, where the duration of support typically ranges from several days to even weeks. Consequently, values of plasma free hemoglobin (ΔHb) may increase to a level where the haptoglobin—a protein that scavenges hemoglobin—becomes depleted, potentially leading to organ damage.^1^, ^2^ Clinically significant hemolysis is assessed by measuring ΔHb concentration, with reported cut‐off values in the literature ranging from 50 to 200 $\mathrm{mg}\cdot L^{-1}$, while levels exceeding 500 $\mathrm{mg}\cdot L^{-1}$ are generally considered indicative of severe hemolysis.^3^ Thus, clinically significant hemolysis can be considered a crucial factor in determining the clinical suitability of a pump, with RPs and centrifugal pumps (CPs) being the two main types used in these procedures.^4^, ^5^

In short‐term applications, such as CPB, no clear difference has been observed between RPs and CPs in terms of hemolysis. A review^6^ analyzed seven comparative studies, of which only one reported worse hemolytic markers for RPs. For a longer‐term use, such as VA‐ECMO, the application of RPs is currently limited to pediatric patients. A meta‐analysis^7^ included registry data from 4 studies with 9111 pediatric patients in total. They proved that the patients supported with CPs are more prone to develop clinically significant hemolysis (18% vs. 4% in case of RPs) and they also seem to have higher in‐hospital mortality. However, it is important to note that the hemolysis induced during clinical procedures in patients supported by both pumps is far from negligible (especially with CPs in VA‐ECMO) and any potential reduction in hemolysis could lower the incidence of clinically significant hemolysis, thereby improving the overall safety of extracorporeal circuits.

In RPs, the occlusion setting is a primary determinant of hemolysis and is generally established during circuit preparation as a predefined operating condition before the initiation of extracorporeal support.^8^ Despite this, they are almost always operated under standard full‐occlusion conditions due to concerns that backflow through a non‐occlusive gap may introduce inaccuracies in flow measurement.^9^ In vitro experiments, however, have demonstrated that under‐occlusive RP settings result in lower levels of hemolysis compared to full occlusion,^9^, ^10^, ^11^ even when the flow rate is reduced by less than 10% in certain cases.^9^ While CPs were found to be superior to both RP configurations,^9^ this outcome may have been confounded by inappropriate under‐occlusion adjustment, as another study reported significantly lower hemolysis in under‐occlusive RP compared with CP.^10^

Although many relevant computational studies exist among the literature, most of them are focused on CPs^12^, ^13^, ^14^ and it appears that their optimization has reached a stage where only minor improvements are possible. In the case of RPs, two completely different computational approaches have been utilized. The first approach provides a more realistic representation, as it captures the complex 3D deformation of the tube arising during compression via two‐way fluid–structure interaction (FSI) simulation.^15^, ^16^ However, these studies present only basic results—such as velocity fields, flow rates, and stresses within the tube without any parameter optimization or hemolysis modeling. This limitation is likely due to the enormous complexity of such simulations, which makes them highly time‐consuming and prone to divergence. Moreover, the under‐occlusive gap sizes considered in these simulations remain well above clinically relevant values, most likely due to convergence issues on the structural side. The second approach is more computationally efficient and better suited for parametric optimization. It considers only a 2D computational fluid dynamics (CFD) model of an RP, incorporating roller‐induced boundary deformation through a dynamic mesh. This deformation is described analytically and implemented into a CFD solver via a user‐defined function (UDF).^17^

Unfortunately, all the aforementioned studies, when employing an under‐occlusive setup, rely on oversimplified boundary conditions (BCs). The inlet and outlet BCs are typically prescribed as zero pressure (i.e., zero pressure gradient along the pump) or as a no‐flux condition. Such BCs, combined with an under‐occlusive setting, result in an unrealistic setup that cannot occur in actual clinical applications. For instance, in VA‐ECMO systems, operating outlet pressures reach up to around 300 mmHg, while the inlet is subject to negative (suction) pressure, resulting in a clear positive pressure gradient across the pump.^18^, ^19^, ^20^ Moreover, the optimization in^17^ focuses on the evaluation of maximum wall shear stresses (WSS), without directly modeling hemolysis (e.g., via a shear‐induced model), which is a nonlinear function of both shear stress and exposure time. This implies that assessing hemolysis solely on the basis of maximum WSS values may be insufficient and could therefore lead to misleading conclusions. For instance, extensive regions with lower shear stress but prolonged exposure time may be overlooked, despite their potentially significant contribution to overall hemolysis. In contrast, implementing a mathematical model that directly calculates the hemolysis allows for quantifying the total hemolysis at the domain outlet, thereby accounting for all hemolysis generated within the pump.

To the best of the authors' knowledge, no existing study combines CFD simulations of an RP with a shear‐induced hemolysis model and experimentally obtained BCs within a clinically relevant range. Such CFD‐based flow analysis can provide detailed insight into local hemodynamics, enabling the estimation of realistic shear stresses and thereby elucidating the mechanisms responsible for hemolysis occurrence. This includes identifying critical regions and phases of the pumping cycle that are particularly prone to hemolysis. More importantly, it enables the quantification of hemolysis levels across different under‐occlusive settings, which could provide a useful reference for establishing a clinically appropriate degree of under‐occlusion.

## 2 METHODS

The study evaluates hemolysis in an in‐house two‐RP across five distinct degrees of under‐occlusion using computational simulations with experimentally obtained BCs. The in‐house RP was designed to enable precise adjustment of the under‐occlusive gap, which was progressively increased across the five investigated settings, each characterized by the corresponding reduction in flow rate relative to full occlusion.^8^, ^9^ To ensure comparability among the different configurations, the same rotational speed and constant peripheral resistance were maintained, with the latter accounting for the influence of other components within an extracorporeal circuit. Consequently, each under‐occlusion setting resulted in different operating pressures and flow rates. Specifically, the methodology is tailored to VA‐ECMO operating conditions, where hemolysis is most significant due to its prolonged duration.

### 2.1 Experimental setup

A schematic illustration of the experimental circuit is shown in Figure 1 left, where the components are listed in the direction of flow as reservoir 1, magnetic‐inductive flow meter 2, inlet pressure sensor 3, in‐house RP with adjustable occlusion 4, outlet pressure sensor 5, and control valve 6. Both pressure sensors operate at a sampling frequency of 200 Hz and are deliberately scaled to clinically relevant inlet and outlet pressures to ensure maximum measurement accuracy. As the working fluid, a water‐glycerol solution was used, with the volume ratio of water to glycerol chosen to match the constant dynamic viscosity of blood under higher shear rates (μ≈3.5cP ^21^). It is acknowledged that, in clinical applications, the circulating fluid is real blood, which exhibits non‐Newtonian behavior. However, at higher shear rates (>100 s^−1^)^21^, blood behaves more like a Newtonian fluid, with an effective viscosity comparable to that used here. Red blood cell damage, in contrast, typically occurs only at much higher shear stresses—above 400 Pa^22^—corresponding to shear rates on the order of $10^{5}$ $s^{-1}$. Moreover, the objective of this study is not to obtain absolute quantitative values but rather to perform a relative comparison among the individual configurations. Therefore, in this context, the use of a glycerin‐water solution is not only sufficient but also advantageous, as working with real blood in an experimental setup introduces significant challenges related to handling (e.g., anticoagulation requirements) and ethical considerations.

**FIGURE 1:**
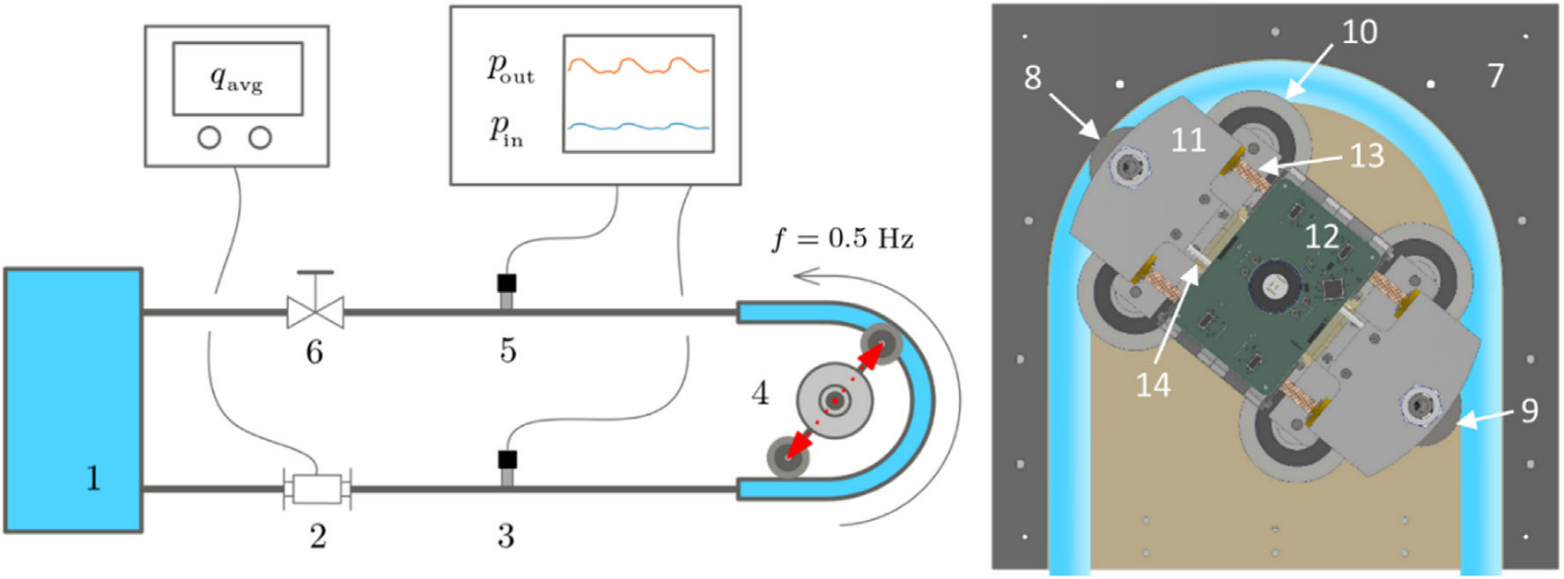
Schematic representation (top view) of the experimental circuit incorporating an in‐house RP with adjustable occlusion (left) and a detailed model of the pump (right). Components: Reservoir 1, magnetic‐inductive flow meter 2, inlet pressure sensor 3, in‐house RP 4, outlet pressure sensor 5, control valve 6, aluminium stator 7, leading roller 8, trailing roller 9, guiding rollers 10, roller carriers 11, printed circuit board 12, lead screws 13, and linear guide 14.

A detailed model of the two‐roller RP 4 is shown in Figure 1 right. It consists of an aluminium stator housing 7 with an inner diameter of 280 mm and a rotor mounted inside. A Tygon tube is positioned between the rotor and stator, with an internal diameter of 19 mm and a wall thickness of 3.2 mm. The rotor features a leading and a trailing roller (8 and 9) with a 180° occlusion angle, as well as four guiding rollers 10 that prevent vertical displacement of the tube. The peristaltic segment between the rollers 8 and 9 delivers a pumping volume of approximately 100 mL. The rotor of the peristaltic pump is driven by a motor mounted underneath, which is not visible in the figure. In the central part of the mounting platform, two pairs of linear stepper motors are installed, each pair controlling a single roller carrier 11. These motors are not visible in the figure, as they are covered by a printed circuit board 12. The stepper motors drive trapezoidal lead screws 13 (4 in total) threaded into the carrier wall. Each stepper motor is independently controlled, and the carriers slide along a linear guide 14 (2 in total) mounted on the platform in the direction of their motion. By rotating the lead screws in either direction, the radial position of the roller carrier—and thus the degree of under‐occlusion—can be precisely adjusted, allowing for highly accurate control and fine‐tuning of occlusion.

Control valve 6 was used to simulate the hydraulic resistance arising from components of extracorporeal VA‐ECMO circuits (e.g., oxygenator and cannulas). As a first step, the pump rotational speed was set to $n=30\;\mathrm{mi}n^{-1}$ (f=0.5 Hz) under standard full‐occlusion condition, yielding a flow rate of $q_{\mathrm{fo}}=6\;l\cdot \mathrm{mi}n^{-1}$. Furthermore, the valve opening was set so that the resulting outlet pressure reached 350 mmHg, ensuring that both flow rates and pressures remained within the clinical range typically reported for VA‐ECMO systems.^18^, ^19^, ^23^ Once this target value was achieved, the valve position remained unchanged throughout the subsequent measurements to maintain a constant hydraulic resistance, reflecting the fixed configuration of a VA‐ECMO circuit. Thereafter, only the occlusion level was altered to establish distinct under‐occlusion conditions.

The tightest under‐occlusion degree was then deliberately adjusted to achieve a 10% flow reduction ($\Delta q_{{\mathrm{rel}}_{1}}$, see Equation (1)), a representative value for clinical under‐occlusion,^9^ resulting in an average flow rate of $q_{{\mathrm{uo}}_{1}}$= 5.4 $L\cdot \mathrm{mi}n^{-1}$. Subsequently, four additional degrees of under‐occlusion were measured to capture the trend across a wider range. The averaged measured pressure and flow values, together with their corresponding gap size used in the CFD simulations for each under‐occlusion setting are summarized in Table 1.

(1) $$∆q_{\mathrm{rel}}\left[\%\right]=\frac{q_{\mathrm{fo}}-q_{uo_{x}}}{q_{\mathrm{fo}}}\cdot 100$$

**TABLE 1:** Individual under‐occlusion settings (uo_*x*_) and their averaged measured values with corresponding gap in 2D CFD simulation.

	*q* [L · min^−1^]	Δ*q*_rel_ [%]	*p*_out_ [mmHg]	*p*_in_ [mmHg]	Gap (CFD) [mm]
uo_1_	5.4	10	243	−15.3	0.125
uo_2_	4.5	25	174	−14.3	0.3
uo_3_	3.8	37	139	−12.4	0.41
uo_4_	3	50	85	−10.7	0.618
uo_5_	2.2	63	55	−12.5	0.9

Since the under‐occlusion gap is challenging to measure accurately, the under‐occlusion setting in the CFD simulation was verified by ensuring that the calculated average velocity matched the measured average velocity (derived from individual $q_{{\mathrm{uo}}_{x}}$). Moreover, all average flow rates measured with magnetic‐inductive flow meter 2 were validated against a standard, robust gravimetric method.

Considering the computational mesh resolution within the under‐occlusive gap, the 10% flow‐reduction case, which already involved a very narrow gap of 0.125 mm in the CFD simulation, was selected as the smallest investigated gap. This, in turn, required extremely fine near‐wall elements to accurately resolve the boundary layer, which is essential for accurate shear stress evaluation and, consequently, reliable hemolysis assessment. Moreover, tuning the under‐occlusion further toward full occlusion would result in only minimal flow reductions, which are difficult to measure reliably.^9^

### 2.2 CFD simulations

The numerical simulations were performed using Ansys Fluent 2024 R2 (ANSYS Inc., USA). A simplified 2D finite volume (FV) model of RP was created based on its actual geometry, illustrated in Figure 2 (left). For the computational domain, only the necessary part of the circuit was considered, defined from the inlet to the outlet pressure sensor. These positions therefore serve as the domain boundaries, where the measured pressure signals are prescribed. It is important to note that, although the control valve—used to set the hydraulic resistance of the circuit to clinically relevant levels—is not explicitly included in the computational domain, its effect is inherently captured in the measured pressure signals prescribed at the boundaries. This crucial aspect has been entirely overlooked in previous computational studies. The remaining boundaries of the domain can be categorized into static and deforming walls. The static wall includes the inlet and outlet segments of the rigid tubing located between the pressure sensors and the pump, as well as the outer wall of the peristaltic tube, whose motion is restricted by the pump stator. In contrast, the deforming wall corresponds to the inner wall of the peristaltic tube, which undergoes a time‐dependent deformation accounting for the roller motion. To prescribe the wall deformation, the method described in Reference [17] was adopted and implemented via a UDF.

**FIGURE 2:**
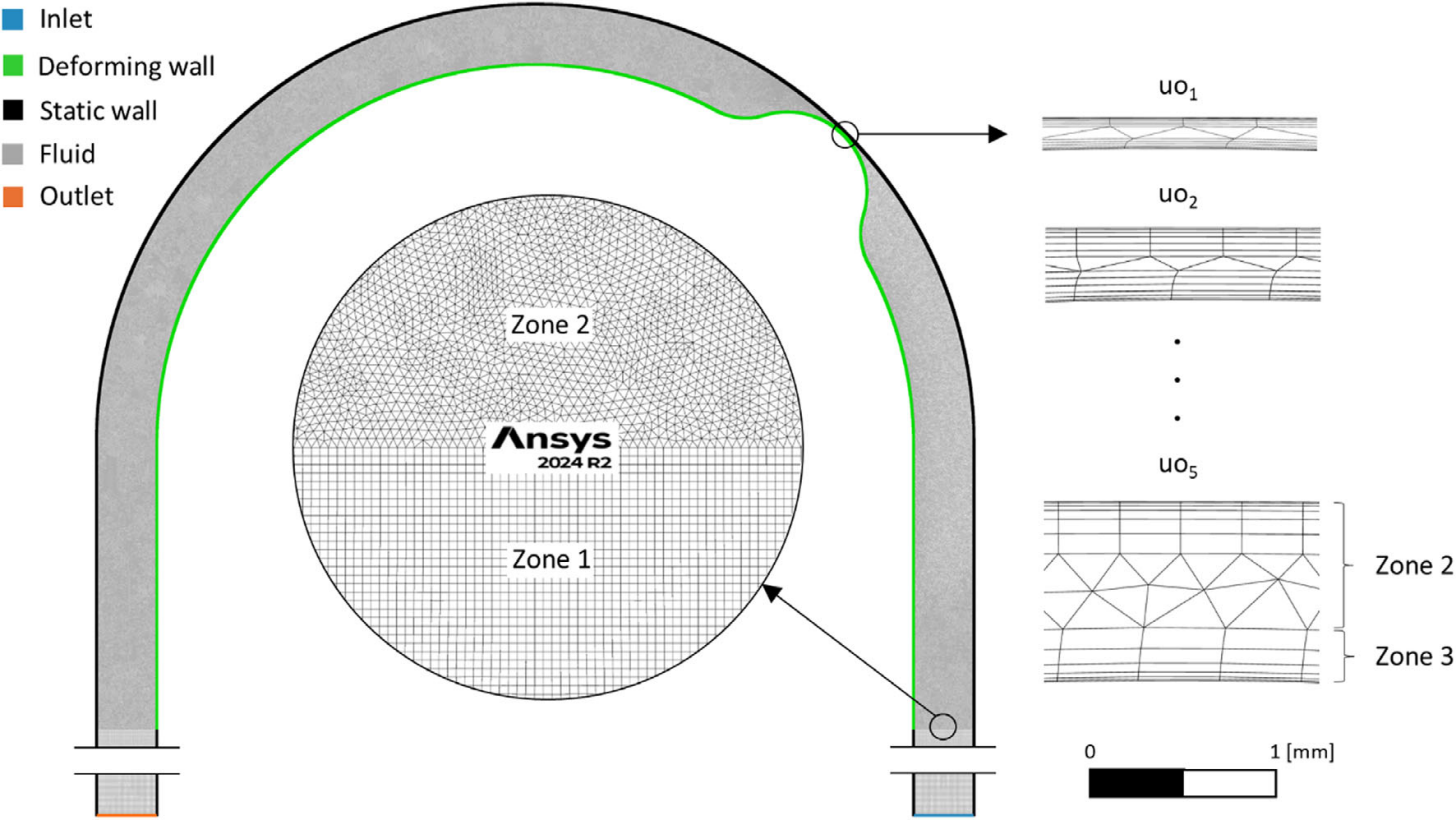
Overview of the computational domain showing its boundaries and zoomed views of individual zones for dynamic meshing, with meshes for progressively increasing under‐occlusive gaps shown on the right.

The computational mesh, consisting of approximately 314,000 elements, was divided into three zones (see zoomed views in Figure 2 right). The first zone corresponds to the non‐deforming region at the inlet and outlet tubing, where structured quadrilateral elements were used to reduce the overall number of elements. The peristaltic segment, which was subject to dynamic meshing, was further divided into two additional zones. The second zone comprises the core of the peristaltic segment together with the prism layer at the static wall and was treated with remeshing and smoothing. The core was meshed with unstructured triangular elements (0.3 mm characteristic size), which are suitable for remeshing. The third zone contains only the prism layer at the deforming wall, representing the remainder of the peristaltic segment, and was treated with smoothing only. This separation allowed the roller‐induced deformation to be accommodated primarily within the triangular core region, where unified remeshing was applied in combination with smoothing, while the near‐wall prism layers were preserved using smoothing only. The prism layers were adjusted to satisfy the near‐wall resolution requirements of the turbulence model used for the simulations, resulting in a first‐layer element height of 0.007 mm (see each mesh gap detail in Figure 2). This value proved sufficient for all under‐occlusive configurations. Further information on the flow regime and the turbulence model employed in the simulations can be found in Appendix B. In addition, a mesh‐independence analysis was performed, confirming the adequacy of the selected mesh for the present application. The corresponding results are provided in Appendix C.

Among the monitored quantities during the simulation, hemolysis required the longest stabilization time, typically reaching convergence in the fifth period, meaning that the solution attained a periodic state in which the results of two consecutive periods were identical. To guarantee sufficient stabilization time and to avoid mesh collapse due to dynamic mesh motion, the smoothing parameters were tuned in preview mesh motion using diffusion functions and their respective parameters. In particular, the boundary distance diffusion function was chosen, as it provided better convergence and dynamic mesh stability than the cell volume function. The diffusion parameter was gradually reduced with decreasing gap size, corresponding to values of 1.7, 1.5, 1.3, 1.2, and 0.5, respectively. This reduction was necessary since, for smaller gaps, the prismatic layer had to be compressed as its height exceeded the actual gap width. All other dynamic mesh parameters were kept at their default settings, with the Implicit Update option enabled.

The fluid was modeled as a Newtonian, incompressible liquid with a dynamic viscosity of μ=3.5 cP and a density of $\rho =998.2\;\mathrm{kg}\cdot m^{-3}$, consistent with the experimental setup. For the hemolysis evaluation, a shear‐induced model was implemented in the computational framework (see Appendix A for details).

#### 2.2.1 Boundary conditions

Measured pressure waveforms were specified at both the inlet and outlet as BCs (see Figure 3). During the measurements, the roller position was recorded, which allowed for proper phase alignment of the BCs with the roller position in the CFD simulation. The pressure profiles are plotted over the interval until a roller completes half a revolution (i.e., one period, T=1 s). To facilitate comparison with clinical data, all values are reported in mmHg. For all under‐occlusion settings, the data reference position was set to the moment when the rollers reached vertical position (see RP position in Figure 3 at t=0 s). At this point, the leading roller is already at its maximum compression of the tube, whereas the trailing roller remains inactive until it starts compressing the tube on the suction side at t=0.31 s, leading to a gradual increase in inlet pressure. The leading roller maintains maximum compression until t=0.5 s, when it begins to release the tube. This is followed by a steep pressure drop in outlet pressure, as the fluid that was on the suction side (exposed to low pressure) is now being discharged by the opposite roller, which now takes over as the leading one. This pressure drop becomes less pronounced with increasing under‐occlusive gap. The roller that has just completed its discharge becomes the trailing one and gradually releases and leaves the tube at t=0.81 s. At t=1 s, the entire process repeats.

**FIGURE 3:**
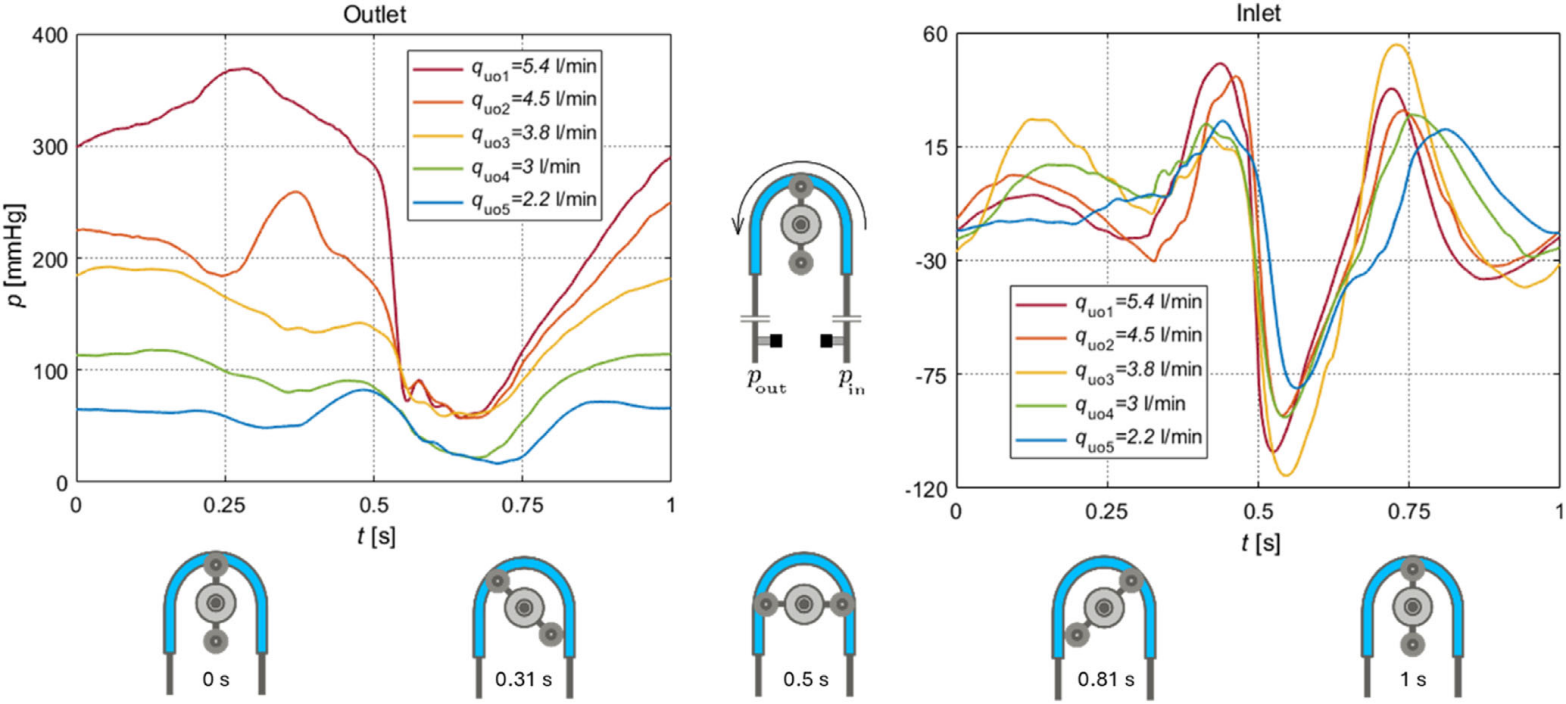
Individual settings of under‐occlusion and their resulting pressure waveforms at the outlet (left) and inlet (right) of the RP, including an illustration of the corresponding RP positions (identical for all settings).

Given that the flow generated by the RP is inherently pulsatile, the simulations were performed in a transient manner. With regard to the roller motion, the authors in Reference [17] suggest choosing the time step according to the time required for the roller to advance by $0.1^{{}^{\circ}}$. In this case, the roller rotates 180° per second, which yields a conservatively rounded value of $t_{s}=0.0005$ s. This value is also considered sufficient with respect to the measured BCs. The BCs for the shear‐induced model of hemolysis were set in Ansys Fluent GUI, where the inlet and outlet BCs were defined as zero values (assuming blood without any initial ΔHb). The scalar flux through the walls was also set to zero, reflecting the impermeability of the peristaltic tubing.

## 3 RESULTS

Since the computational simulations are transient, several pumping cycles were computed to allow the solution to stabilize. All presented results were therefore evaluated starting from the fifth period, at which periodic and stable behavior was achieved. To bring the results to clinically and experimentally comparable levels, the HI_*L*_ was recalculated according to Equation (2) and expressed in percentages, as is common in clinical and in vitro studies.

(2) $$\mathrm{HI}\left[\%\right]=\left(HI_{L}^{\beta }\right)\cdot 100$$

Given that the HI is a passive scalar advected by the velocity field, displaying velocity contours alongside would be redundant, as both fields share essentially the same flow‐driven dynamics.

Therefore, the HI contours are sufficient for general flow analysis. The hemolysis contours were evaluated throughout the entire pumping cycle for each occlusion setting. For clarity, Figure 4 presents only selected representative configurations and time points that capture the main observed flow and hemolysis characteristics. The uo_1_ setting was included as a reference condition, uo_2_ was selected because it exhibited the highest integrated hemolysis over the pumping cycle, and uo_4_ was chosen as the first configuration in which a pronounced decrease in this quantity was observed. Moreover, all contours are shown using the same HI scale to enable direct comparison. Although uo_1_ exhibits the highest local HI values ($HI_{\max}\approx 1.8\%$), the scale maximum is set according to the maximum observed in uo_2_ ($HI_{\max}\approx 0.8\%$) to ensure that differences among the settings remain visually distinguishable, including the much lower value observed for uo_4_ ($HI_{\max}\approx 0.12\%$).

**FIGURE 4:**
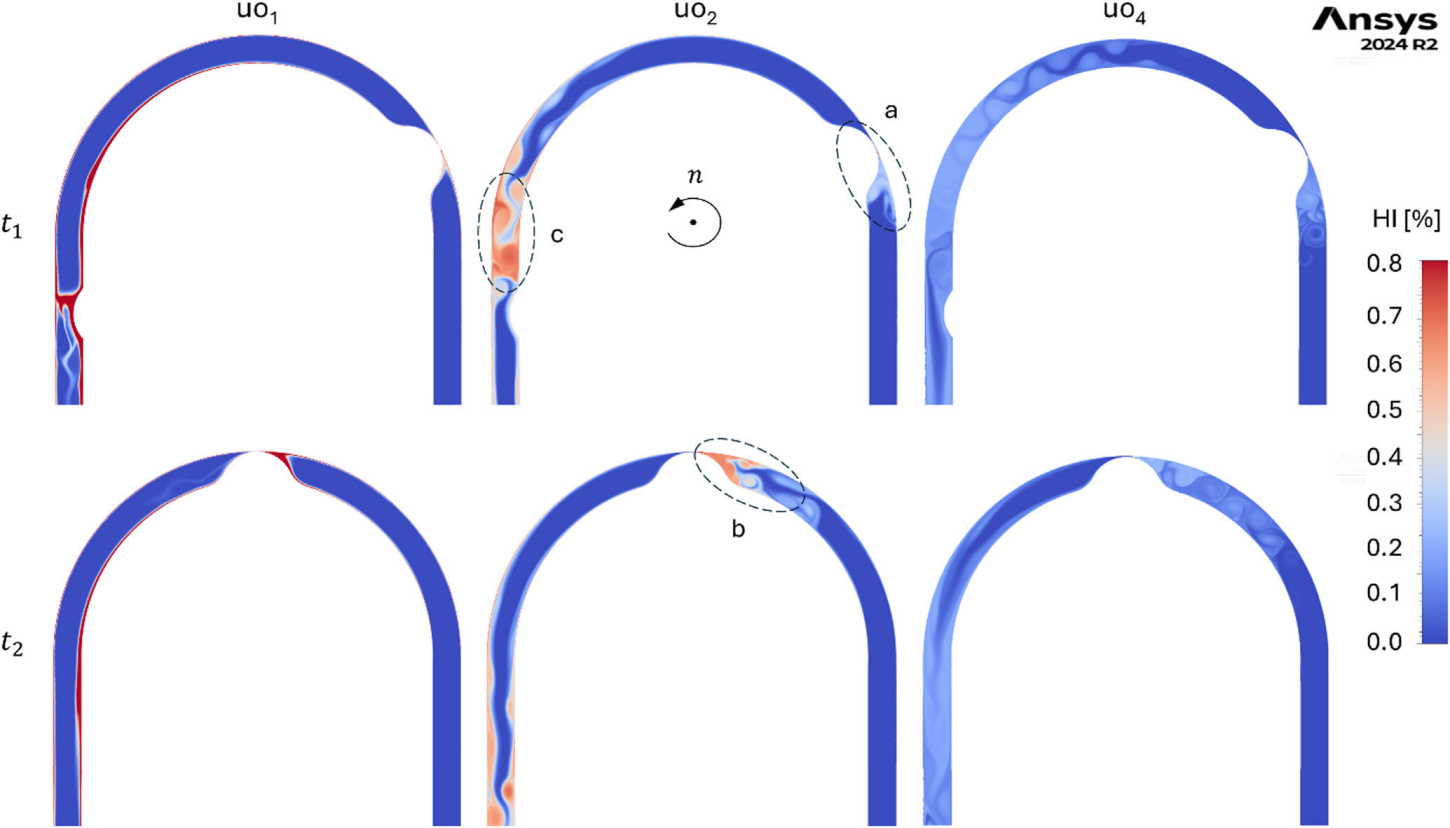
Contours of the hemolysis index (HI) for three representative degrees of under‐occlusion settings (uo_1_, uo_2_, and uo_4_), arranged from the tightest to the loosest setting and shown at two selected time instants, $t_{1}$ and $t_{2}$. Dashed outlines indicate key regions behind the roller, with (a) showing backflow jet and vortex formation, (b) hemolysis progression, and (c) accumulated hemolysis over the pumping cycle. The arrow labeled n indicates the direction of rotation.

One common characteristic—partially predictable based on physical reasoning—was identified. The results for all under‐occlusive configurations clearly show the formation of a reverse velocity jet within the under‐occlusive gap, resulting from fluid being driven toward the lower pressure at the inlet (see also Figure 5, left). This manifests as a local increase in hemolysis in the region behind the leading roller, as shown in the top contours in Figure 4, where the corresponding region is highlighted by the dashed outline labeled (a). Since the jet persists throughout the compression phase, hemolysis progressively accumulates in this region (see bottom contours in Figure 4, region (b)). In the first case (uo_1_), the flow remains overall stable and moderate throughout the entire cycle, which can be explained by the fact that this configuration represents a very tight under‐occlusive setting with the smallest gap, allowing only a minimal portion of the flow to pass through it, which results in a weaker and stable jet. For the other settings (uo_2_ and uo_4_), the reverse jet is much stronger, which leads to its instability and the formation of vortices behind the roller. However, in the case of uo_4_, the gap becomes sufficiently large such that, relative to the other settings, the resulting hemolysis is substantially reduced, and thus the flow characteristics are less apparent. The total hemolysis generated over the entire compression phase can again be observed in the top contours in Figure 4, but this time in the region behind the opposite roller, highlighted as region (c).

**FIGURE 5:**
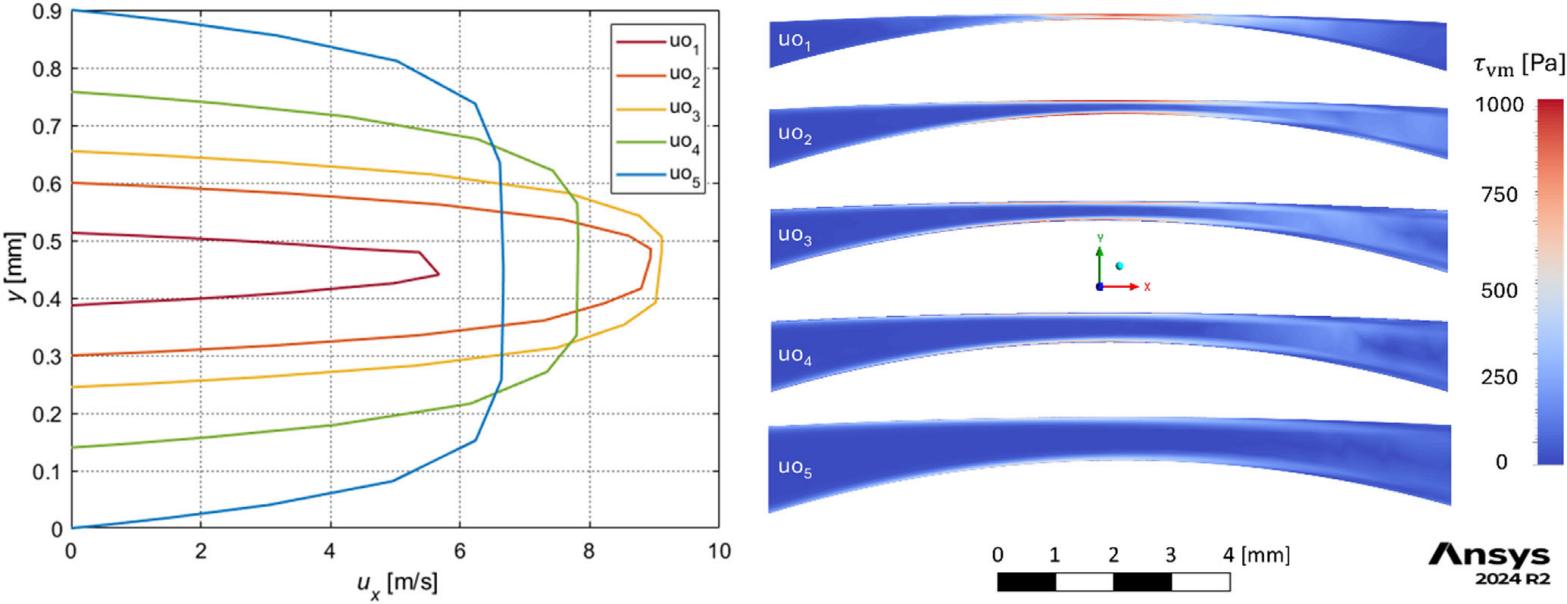
Velocity profiles of the backflow jet (left) and the corresponding $\tau_{\mathrm{vm}}$ contours (right) for the individual under‐occlusive gaps, shown at the moment when a roller is in the vertical position.

Although the flow characteristics differ between the individual under‐occlusive settings, it was consistently observed throughout the entire period that the majority of hemolysis originates within the under‐occlusive gap and its vicinity, corresponding to the region of the high‐velocity jet, where $\tau_{\mathrm{vm}}$ values remain substantially higher than in the rest of the fluid domain (see Figure 5, right). This is further enhanced by the exposure time $t_{\exp}$ of the compression phase, which, being the phase responsible for generating flow, spans the entire cycle. Therefore, the flow characteristics in the region behind the roller primarily determine how the HI is subsequently transported throughout the domain, but they do not significantly contribute to its generation.

To quantify the hemolysis generated over a full half‐rotation of the roller, HI was evaluated near the RP outlet as a velocity‐weighted average according to Equation (3), where the normal velocity component is defined as $u_{n}=u_{i}n_{i}$. Velocity‐weighting is essential, as local flow velocities vary across the outlet cross‐section and directly affect the residence time of HI.^23^

(3) $${\mathrm{HI}}_{\text{out}}=\frac{\int_{A}u_{n}\mathrm{HI}\;\mathrm{dA}}{\int_{A}u_{n}\;\mathrm{dA}}$$

The resulting values were then compared across the five under‐occlusion settings (see Figure 6, left). For uo_1_ and uo_2_, a pronounced increase in $HI_{\text{out}}$ can be observed around t≈0.75 s, which corresponds to the time when the hemolysis accumulated during the compression phase reaches the location where $HI_{\text{out}}$ is evaluated. A similar, although less pronounced, increase is also observed for uo_3_. In the case of uo_1_, this increase appears as a sharp peak, indicating that hemolysis is generated predominantly just behind the roller. The peak also occurs earlier due to the higher flow rate. In contrast, for uo_2_, and even more clearly for uo_3_, the stronger backflow jet and associated swirling flow transport elevated HI farther along the tube, resulting in a broader spatial distribution and consequently spreading the increase in $HI_{\text{out}}$ over a longer portion of the cycle. For uo_4_ and uo_5_, the $HI_{\text{out}}$ level is much lower, as the under‐occlusive gap is considerably wider. Moreover, $HI_{\text{out}}$ becomes increasingly distributed over the entire pumping cycle, with the temporal profile becoming relatively flat for uo_5_. This behavior results from the substantially stronger backflow jet, which redistributes elevated HI across nearly the entire pump domain. The resulting redistribution for the representative settings can be visually observed in the region behind the roller during tube release (see region (c) in each top contour of Figure 4).

**FIGURE 6:**
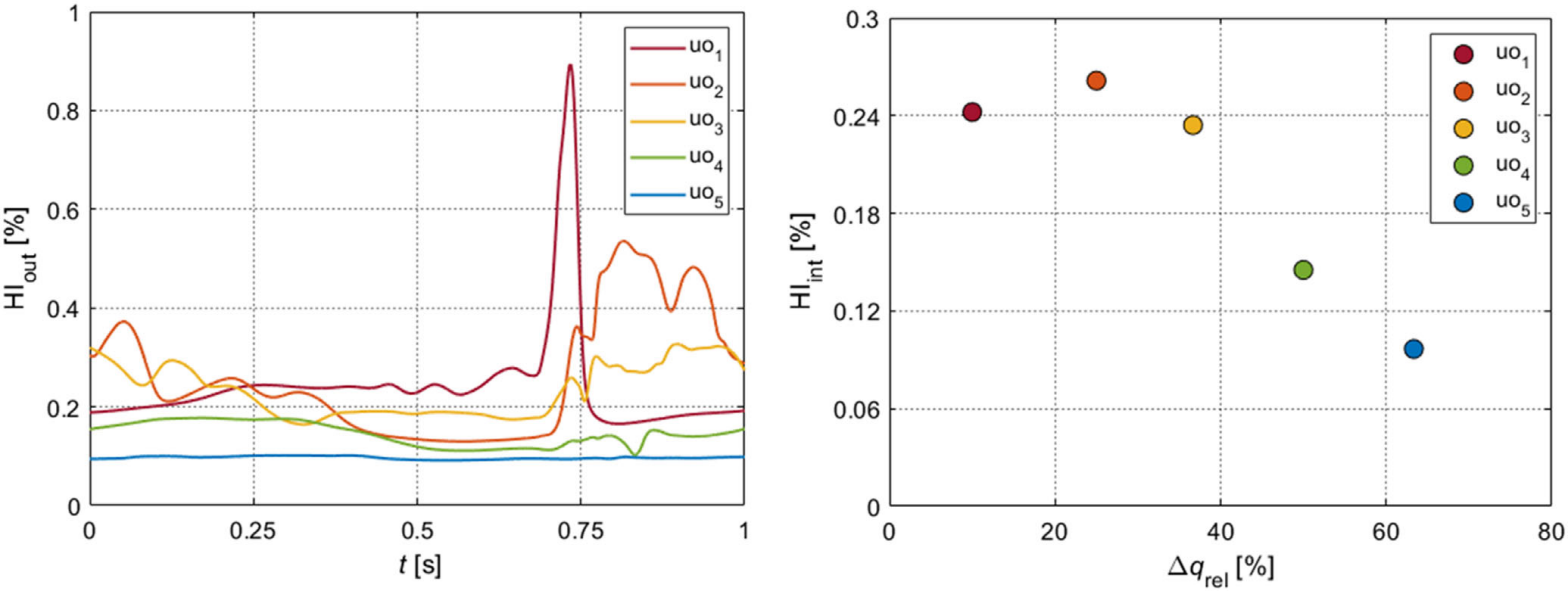
Generated hemolysis over the entire cycle for each under‐occlusion setting collected as velocity‐weighted average (left), and the corresponding integrated values plotted in dependence on the flow rate reduction due to under‐occlusion, $\Delta q_{\mathrm{rel}}$ (right).

Finally, $HI_{\text{out}}$ values were time‐averaged to obtain the total amount of HI generated per period for each under‐occlusion setting (Equation (4)). These values were then plotted in dependence on the corresponding flow rate reduction $\Delta q_{\mathrm{rel}}$ (see Equation (1) and Figure 6 right).

(4) $${\mathrm{HI}}_{\mathrm{int}}=\frac{1}{T}\int_{0}^{T}HI_{\text{out}}\mathrm{dt}$$

The results show that the three tighter under‐occlusion settings, uo_1_–uo_3_, yield comparable levels of hemolysis, forming a relatively broad plateau despite the progressive increase in the under‐occlusive gap and the associated reduction in flow rate. Among these settings, uo_2_ exhibits the highest hemolysis, while uo_1_ and uo_3_ remain at very similar levels even though they represent substantially different degrees of under‐occlusion. This finding suggests that tighter under‐occlusion may be beneficial not only from the perspective of flow performance, but also in terms of hemolysis. It may further indicate that hemolysis does not necessarily follow a decreasing trend with increasing under‐occlusion, as is the case for the flow rate. This behavior can be attributed to the competing effects of the under‐occlusive gap size and the resulting backflow jet. While a wider gap alone would theoretically reduce shear stress and hemolysis, it also allows a larger volume of blood to pass through the gap and promotes the development of a stronger reverse jet, which increases local shear stresses and may lead to comparable or even more adverse hemolytic conditions (see uo_1_–uo_3_ in Figure 5, right). Nevertheless, this behavior appears to be limited to the tighter under‐occlusion settings, since hemolysis decreases markedly for uo_4_ and uo_5_. This reduction, however, comes at the expense of a significantly lower flow rate.

## 4 DISCUSSION

In this study, a time‐dependent pressure gradient was prescribed in the hemodynamic 2D CFD simulation of the RP, using experimentally measured inlet and outlet pressure waveforms. Its magnitude was adjusted by a control valve, calibrated under fully occlusive conditions to reflect clinically relevant conditions. This gradient is one of the primary determinants of the backflow intensity in the under‐occlusive gap—that is, a stronger pressure gradient (e.g., a more closed valve) results in a stronger reverse jet and consequently lower flow performance. This demonstrates a characteristic feature of under‐occlusive RPs, which, unlike fully occlusive configurations, are no longer afterload independent. However, this requires the under‐occlusive gap in the simulation to be set in correspondence with the measured pressure BCs, which, in this study, was achieved by validating the calculated velocity against flowmeter measurements. For this reason, an in vitro circuit is considered to be a fundamental part of the methodology. In contrast, existing computational studies^15^, ^16^, ^17^ assume a zero pressure gradient across the pump, even for different under‐occlusion settings, which, in the absence of an experimental circuit, appears to be the only viable option for the reasons discussed above. However, this oversimplification neglects clinically relevant conditions and therefore yields results that are not representative of real operating scenarios (e.g., in VA‐ECMO systems).

### 4.1 Limitations

The in‐house RP used in this study was designed for multipurpose use and thus does not fully match the dimensional specifications of RPs currently employed in clinical practice, especially in terms of peristaltic tube size. However, key aspects were adjusted to ensure clinical relevance. In particular, the peristaltic tube pumping volume of the two‐roller configuration is approximately 100 mL, which is several times higher than that of standard clinical RPs. Therefore, the rotational speed had to be reduced to achieve clinically relevant VA‐ECMO flow rates. Importantly, the larger pumping volume results not only from the larger tubing diameter but also from the longer peristaltic tubing segment associated with the larger stator. Even though the latter increases the pumping volume, it has a less direct influence on the flow regime than the tubing diameter, which represents the relevant characteristic length.

To assess whether the larger tubing diameter results in a substantially different flow regime, a Reynolds‐number comparison was performed. The same fluid properties as those employed in the CFD simulations were assumed for both tubing configurations. At a given flow rate, the Reynolds number is inversely proportional to the tubing diameter. Using the highest investigated flow rate of 5.4 L/min, Reynolds numbers of approximately 1800 and 2700 are obtained for the 19 mm tubing used in the present experimental setup and the 12.7 mm (1/2‐inch) tubing reported for roller‐pump extracorporeal circuits,^9^, ^19^, ^24^ respectively. These values are of the same order of magnitude and both lie near the commonly cited transitional range. This comparison suggests that the dimensional differences do not induce fundamentally different flow regimes in the two systems. Therefore, although some impact on the absolute hemolysis values can be expected, the observed wide range of under‐occlusion over which hemolysis remained relatively unchanged (see Figure 6, right) is likely to remain relevant also for the smaller dimensions typical of clinical RPs.

The developed methodology for hemolysis evaluation relies on 2D CFD simulations, and thus the tube compression is idealized, particularly for tighter under‐occlusion settings, where, in reality, blood can additionally flow through the bent regions of the tube, forming narrow residual channels on each side. Furthermore, hemolysis is evaluated using a shear‐induced model, supported by experimental measurements conducted with blood‐like solution. Owing to these limitations, the results are intended primarily for relative comparison between the investigated settings rather than as absolute predictions of hemolysis. Given the absence of a clearly defined hemolysis threshold of $\tau_{\mathrm{vm}}$, no specific limit was imposed in these simulations. Nevertheless, comparing the calculated $\tau_{\mathrm{vm}}$ values in the under‐occlusive gap for each setting with the threshold range reported in the literature (100–250 Pa)^25^ indicates that these values remain well above this range for most of the cycle. Therefore, imposing such a threshold would not significantly affect the results or the conclusions drawn from them. To further validate the hemolysis predictions, additional experiments using heparinized porcine or human blood in the experimental circuit would be required. Such validation would involve measuring plasma‐free hemoglobin as a direct marker of hemolysis and comparing the experimental results with the calculated HI values. Given the 2D formulation and the empirical Eulerian hemolysis model, this comparison would primarily help establish a correlation between the predicted and experimentally measured hemolysis rather than demonstrate absolute predictive accuracy.^26^

## 5 CONCLUSION

To this day, the degree of under‐occlusion in RPs remains a topic of considerable interest due to its potential to decrease hemolysis, particularly in prolonged applications such as VA‐ECMO. This article presents a comprehensive methodology for hemolysis evaluation in under‐occlusive RPs, combining 2D CFD simulations with experimental data obtained under clinically relevant conditions. Five distinct under‐occlusive settings were investigated by progressively increasing the under‐occlusive gap, with each configuration defined by its corresponding flow rate reduction relative to full occlusion. Although based on a comparative and highly mechanistic approach, the results indicate that tuning the pump toward a higher degree of under‐occlusion does not necessarily decrease hemolysis. On the contrary, when compared with uo_2_, the tightest setting uo_1_ with a 10% flow reduction appears to be beneficial not only in terms of flow performance but also in terms of hemolysis. More specifically, uo_2_ exhibits approximately 7% higher hemolysis than uo_1_ while also showing a 15% greater loss in flow rate. Interestingly, uo_3_, with a 37% flow reduction, remains at a hemolysis level very similar to uo_1_, indicating that hemolysis stays relatively unchanged across a relatively broad range of under‐occlusion settings despite the progressive increase in gap size. The observed behavior can be attributed to the competing effects of the under‐occlusive gap size and the resulting backflow. While increasing the gap alone would tend to lower shear stresses and hemolysis, it inevitably allows a larger amount of blood to pass through the under‐occlusive gap, promoting the formation of a stronger backflow jet that may lead to comparable or even more adverse hemolytic conditions. As the degree of under‐occlusion increases further in uo_4_ and uo_5_, the advantages of the wider gap prevail and hemolysis decreases markedly. This reduction, however, comes at the expense of a substantial loss in flow performance. Even though these values are specific to the investigated pump geometry, the simulations in the present study were experimentally informed and performed under realistic operating conditions within a clinically relevant range. This extends previous computational investigations by enabling a systematic comparison of different under‐occlusion settings under conditions representative of extracorporeal systems and provides new insight into the interplay between under‐occlusion, flow performance, and hemolysis.

## AUTHOR CONTRIBUTIONS

**Jiří Kohút:** Writing – original draft; methodology; data curation; conceptualization. **Jiří Jagoš:** Methodology; conceptualization. **Pavel Suk:** Conceptualization. **Martin Formánek:** Methodology. **Jiří Burša:** Writing – review and editing; supervision.

## CONFLICT OF INTEREST STATEMENT

The authors declare that a patent application (PV 2025‐126) related to the mechatronic control of occlusion in the in‐house roller pump has been filed.

## Figures and Tables

**FIGURE A1:**
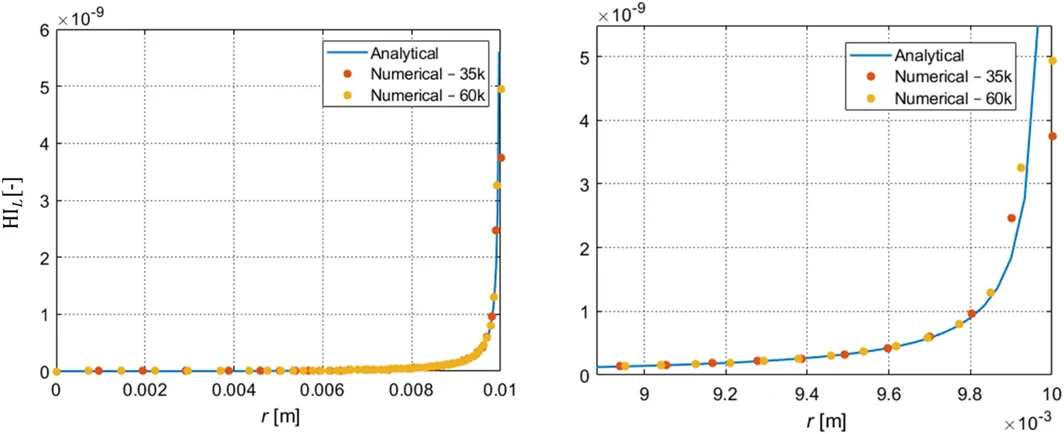
Comparison between analytical and numerical solutions in Ansys Fluent with two different computational meshes. Overall agreement can be observed (left), verifying the correct implementation, while the zoom near the wall (right) highlights the importance of boundary layer refinement.

**FIGURE B1:**
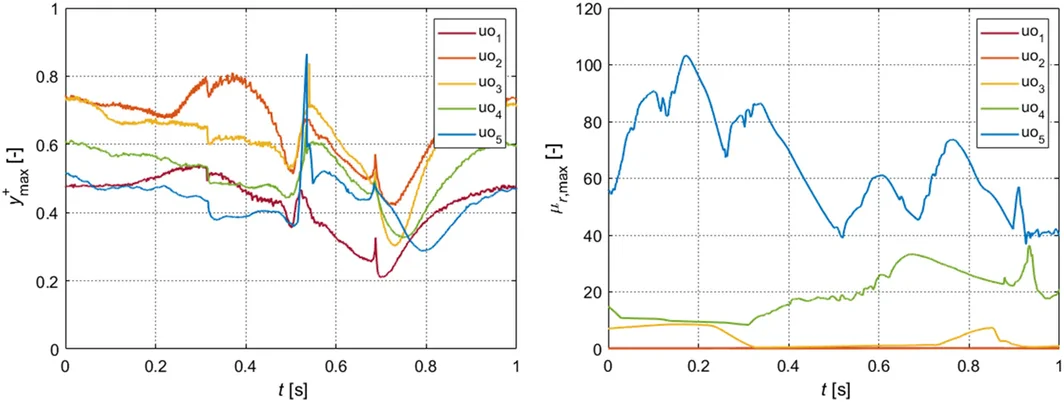
Monitored maximum $y^{+}$ over the entire period for each under‐occlusion setting (left), confirming that all cases satisfy $y^{+}\leq 1$, and the turbulent viscosity ratio $\mu_{r}$ (right), indicating that uo_1_ and uo_2_ remain within the laminar regime ($\mu_{r}$ close to zero), whereas uo_3_‐uo_5_ are progressively more prone to turbulence.

**FIGURE C1:**
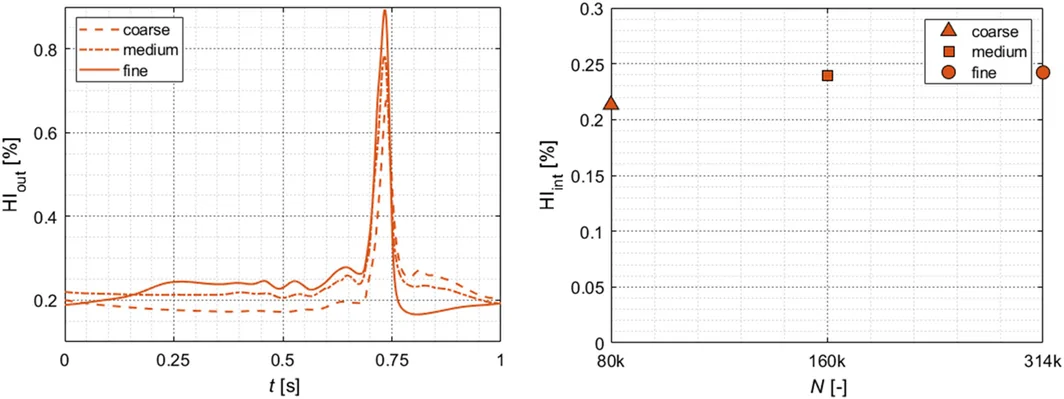
Mesh‐convergence results for the uo_1_ configuration using the coarse, medium, and fine meshes, showing the temporal evolution of ${\mathrm{HI}}_{\text{out}}$ (left) and the corresponding integrated hemolysis index, HI_int_ plotted against the total number of mesh elements *N*.

## Data Availability

The data that support the findings of this study are openly available in Zenodo at https://zenodo.org, reference number DOI: 10.5281/zenodo.17532035.

## APPENDIX A IMPLEMENTATION OF HEMOLYSIS MODEL

To analyze blood damage in cardiovascular devices, either CFD or experimental in vitro measurements are typically employed. Over the last two decades, significant progress in CFD has made it an effective tool for predicting and analyzing hemolysis. Experimentally, the so‐called hemolysis index (HI) is determined from the ratio of plasma‐free hemoglobin (ΔHb) to the total concentration of hemoglobin (Hb). One of the most widely used empirical models is expressed by Equation (A1), also called the power‐law model, where the HI is a non‐linear function of scalar shear stress $\tau_{\mathrm{vm}}$ and exposure time $t_{\exp}$. The real shear conditions (described by a stress tensor) acting on an erythrocyte are represented by a scalar shear stress $\tau_{\mathrm{vm}}$, based on the Von mises criterion with second invariant of the tensor, which is relevant for erythrocyte damage (see Equation (A1)).

(A1)
$$\mathrm{HI}=\frac{\mathrm{\Delta Hb}}{\mathrm{Hb}}=C\tau_{\mathrm{vm}}^{\alpha }t_{\exp}^{\beta }\;\tau_{\mathrm{vm}}=\sqrt{-3II_{\tau }}$$

The model constants 𝐶, α, and 𝛽 were obtained from experimental measurements on human erythrocytes ($C=3.63\cdot 10^{-7}$, α=2.416, β=0.785).^25^ In this formulation, the hemolysis index is presented in a nondimensional form, although it is also commonly expressed as a percentage. The Eulerian power‐law model of hemolysis was linearized in time and incorporated into the scalar transport equation (Equation (A2)) as its source term ($HI_{L}=HI^{1/\beta }$).

(A2)
$$\rho \frac{\mathrm{\partial H}I_{L}}{\partial t}+\rho u_{i}\frac{\partial {\mathrm{HI}}_{L}}{\partial x_{i}}=\rho C^{\frac{1}{\beta }}\tau_{\mathrm{vm}}^{\frac{\alpha }{\beta }}$$

Here, $u_{i}$ denotes the 𝑖th component of the velocity vector, $x_{i}$ is the corresponding spatial coordinate, and ρ is density. Both the hemolysis source term and scalar shear stress were implemented in ANSYS Fluent via UDFs, while the setup of the scalar transport equation was carried out through the Ansys Fluent GUI. The implementation was successfully verified against an analytical solution (see Equation (A3)) for a simplified case involving steady, laminar flow with a parabolic profile in a straight 2D tube matching the diameter of the peristaltic tubing—following a verification procedure similar to that described in Reference [27]. The parabolic profile is imposed at the inlet BC to facilitate validation, resulting in linear dependency of $\tau_{\mathrm{vm}}$ across the radius r.

(A3)
$$HI_{L}=C^{\frac{1}{\beta }}\tau_{\mathrm{vm}}^{\frac{\alpha }{\beta }}\frac{z}{v_{r}}$$

Here, z denotes the axial coordinate and $v_{r}$ is the axial velocity in the radial coordinate *r*. Figure A1 (left) shows a comparison between analytical and numerical results obtained in Ansys Fluent, along with a mesh independence test. Differences in hemolysis values appear only in the near‐wall region, where the velocity gradient is captured more accurately with increasing mesh resolution and, according to Equation (A3), the analytical solution tends to infinity (see zoom in Figure A1, right). Thus, an appropriate near‐wall resolution, sufficient to resolve the boundary layer, may play a decisive role in hemolysis evaluation. Nevertheless, the numerical solution reproduces the expected analytical behavior, and the implementation can therefore be considered verified.

It is worth noting that the Eulerian power‐law model is limited by the fact that its constants were derived from steady, laminar flow experiments, where erythrocytes were exposed to constant shear stresses.^25^ In contrast, the flow in RPs is transient, and the original experiments were conducted under upper limits of τ≤250 Pa and $t_{\exp}\leq 700$ ms. This likely explains why the model tends to overestimate HI compared with experimental results. Nevertheless, it shows a strong correlation and is therefore well‐suited for relative comparisons of different devices.^26^ For this reason, the Eulerian shear‐induced hemolysis model remains one of the most widely used approaches for comparing hemolysis across varying operating conditions, even under unsteady flow and with the k−ω SST turbulence model.^12^, ^13^, ^14^

## APPENDIX B COMPUTATIONAL MODELS AND FLOW REGIME

It is well known that jet‐like phenomena, observable in an under‐occlusive gap, can become rapidly unstable, leading to vortex formation and potential transition to turbulence. In particular, the backflow jet persists throughout the entire cycle, as the high‐ and low‐pressure sides remain connected. As a result, it can promote flow instability downstream of the roller and disrupt the local flow field. Therefore, it is reasonable to assume that the flow might not be strictly laminar. To account for this, the Reynolds‐averaged Navier–Stokes (RANS) equations with Boussinesq hypothesis were used for the problem solution, where the turbulent viscosity $\mu_{t}$ is introduced as a property of the flow. Since the flow regime is assumed to be neither laminar nor fully turbulent, the transitional k−ω SST model with low‐*Re* correction was selected, as it enables damping of turbulent viscosity $\mu_{t}$ and provides a more realistic description of the transitional behavior.

In our previous simulations of transient flow in compliant and rigid tubes,^28^ it was observed that this model was capable of simulating both flow regimes. Specifically, when the flow was laminar, the results obtained with the laminar model and the low‐*Re* k−ω SST model were identical, as $\mu_{t}$ yielded zero values. This model requires $y^{+}\leq 1$ at the wall, which was verified by monitoring the maximum wall $y^{+}$ value (see Figure B1 left). The dimensionless wall distance $y^{+}$ is defined according to Equation (B1), where the friction velocity is given by $u_{\tau }=\sqrt{\tau_{w}/\rho }$ with $\tau_{w}$ being the wall shear stress, and y is the wall‐normal distance to the center of the first computational cell.

(B1)
$$y^{+}=\frac{\rho u_{\tau }y}{\mu }$$

In laminar conditions, Reynolds shear stresses are negligible compared to the actual viscous shear stresses, which corresponds to a very low turbulent viscosity ratio $\mu_{r}$, defined as the ratio of turbulent viscosity $\mu_{t}$ to molecular dynamic viscosity μ (see Equation (B2)).

(B2)
$$\mu_{r}=\frac{\mu_{t}}{\mu }$$

To assess whether the flow regime reached transition‐level values ($\mu_{r}$ >100) or remained dominated by molecular viscosity ($\mu_{r}$≪1), the maximum $\mu_{r}$ value over the entire period was evaluated for each under‐occlusion setting (see Figure B1 right). It can be clearly observed that the tighter under‐occlusion settings (uo_1_ and uo_2_) exhibit values close to zero, indicating laminar flow. In contrast, uo_3_–uo_5_ exhibit progressively increasing maximum values, with uo_5_ reaching values close to the transition threshold and representing the most turbulence‐prone setting.

## APPENDIX C MESH INDEPENDENCE ANALYSIS

A quantitative three‐grid convergence analysis was performed to assess the influence of spatial discretization on the predicted hemolysis. The uo_1_ configuration was selected because its narrow under‐occlusive gap imposed the most stringent requirements on mesh resolution among the investigated configurations. The coarse, intermediate, and fine meshes consisted of approximately 80,000, 158,000, and 314,000 elements, respectively. The integrated hemolysis index, ${\mathrm{HI}}_{\mathrm{int}}$, was selected as the quantity of interest. This integrated quantity accounts for hemolysis generated throughout the pump over the entire cycle and was therefore considered more representative for the mesh‐convergence assessment than a local instantaneous value. Figure C1 presents the temporal evolution of ${\mathrm{HI}}_{\text{out}}$ (left), together with the resulting ${\mathrm{HI}}_{\mathrm{int}}$ values and the corresponding total numbers of mesh elements N (right).

The convergence analysis was performed using the Richardson extrapolation and Grid Convergence Index (GCI) procedure.^29^ The characteristic grid spacing was determined from the number of elements in the two‐dimensional computational domain. The refinement ratios between consecutive mesh levels were nearly identical at approximately r=1.4, exceeding the commonly recommended minimum value of 1.3. The convergence ratio was of 0.01, indicating monotonic convergence as its value was greater than zero. The Richardson extrapolation procedure yielded an observed order of accuracy of p=6.78 and a Richardson‐extrapolated value of ϕ=0.2425%. The relatively high observed order p is likely influenced by the use of an integrated quantity, for which local discretization errors may partially compensate over the pumping cycle. The approximate relative difference between the fine and intermediate meshes was $e_{a}=1.06\%$, while the relative difference between the fine‐grid result and the Richardson‐extrapolated value was $e_{\mathrm{ext}}=0.11\%$. Using a safety factor of 1.25, the resulting GCI was 0.14%, indicating a very low remaining discretization uncertainty in the evaluated integrated hemolysis index. The fine mesh was therefore considered sufficiently resolved for the comparative analysis presented in this study.
